# Two-Dimensional Metal Halides for X-Ray Detection Applications

**DOI:** 10.1007/s40820-023-01118-1

**Published:** 2023-05-20

**Authors:** Yumin Li, Yutian Lei, Haoxu Wang, Zhiwen Jin

**Affiliations:** School of Physical Science and Technology and Lanzhou Center for Theoretical Physics and Key Laboratory of Theoretical Physics of Gansu Province, Lanzhou University, Lanzhou, 730000 People’s Republic of China

**Keywords:** Two-dimensional perovskite, High stability, Ion migration, Charge transport, X-ray Detector

## Abstract

Metal halide perovskites have recently emerged as promising candidates for the next generation of X-ray detectors due to their excellent optoelectronic properties. Especially, two-dimensional (2D) perovskites afford many distinct properties, including remarkable structural diversity, high generation energy, and balanced large exciton binding energy. With the advantages of 2D materials and perovskites, it successfully reduces the decomposition and phase transition of perovskite and effectively suppresses ion migration. Meanwhile, the existence of a high hydrophobic spacer can block water molecules, thus making 2D perovskite obtain excellent stability. All of these advantages have attracted much attention in the field of X-ray detection. This review introduces the classification of 2D halide perovskites, summarizes the synthesis technology and performance characteristics of 2D perovskite X-ray direct detector, and briefly discusses the application of 2D perovskite in scintillators. Finally, this review also emphasizes the key challenges faced by 2D perovskite X-ray detectors in practical application and presents our views on its future development.

## Highlights

- The classification of 2D perovskite is summarized, and the preparation methods of 2D perovskite according to the requirements of X-ray detection materials are introduced.
- We analyzed the advantages and insufficiency of different devices and introduced improvement measures, including ion migration, charge transfer performance, stability, and 2D/3D heterojunctions.
- Finally, we introduced the potential preponderances of 2D perovskite in the scintillation detection field; meanwhile, the main challenges facing the practical application of 2D perovskite X-ray detectors are analyzed.

## Graphical abstract

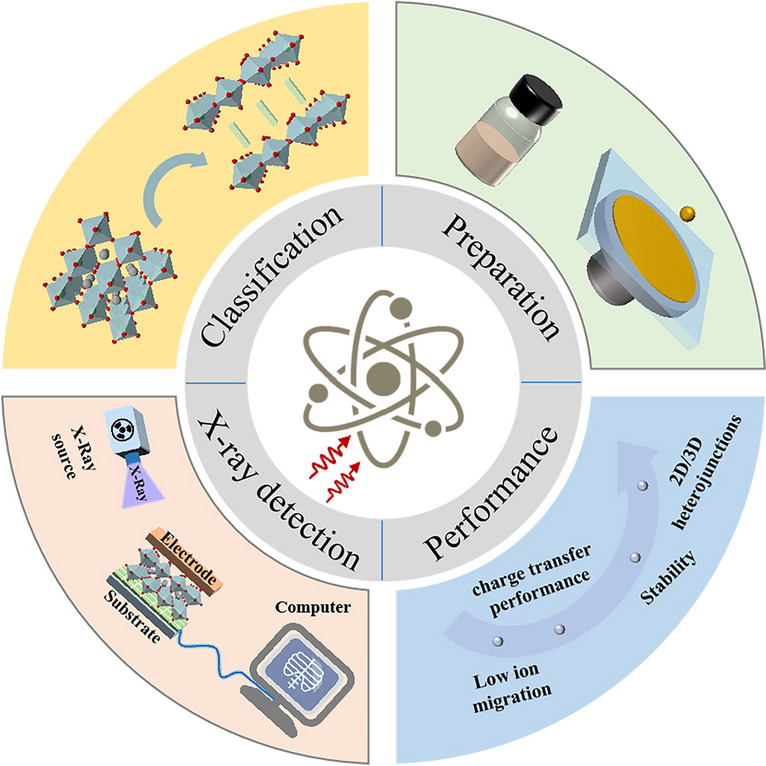

## Introduction

**Scheme 1:**
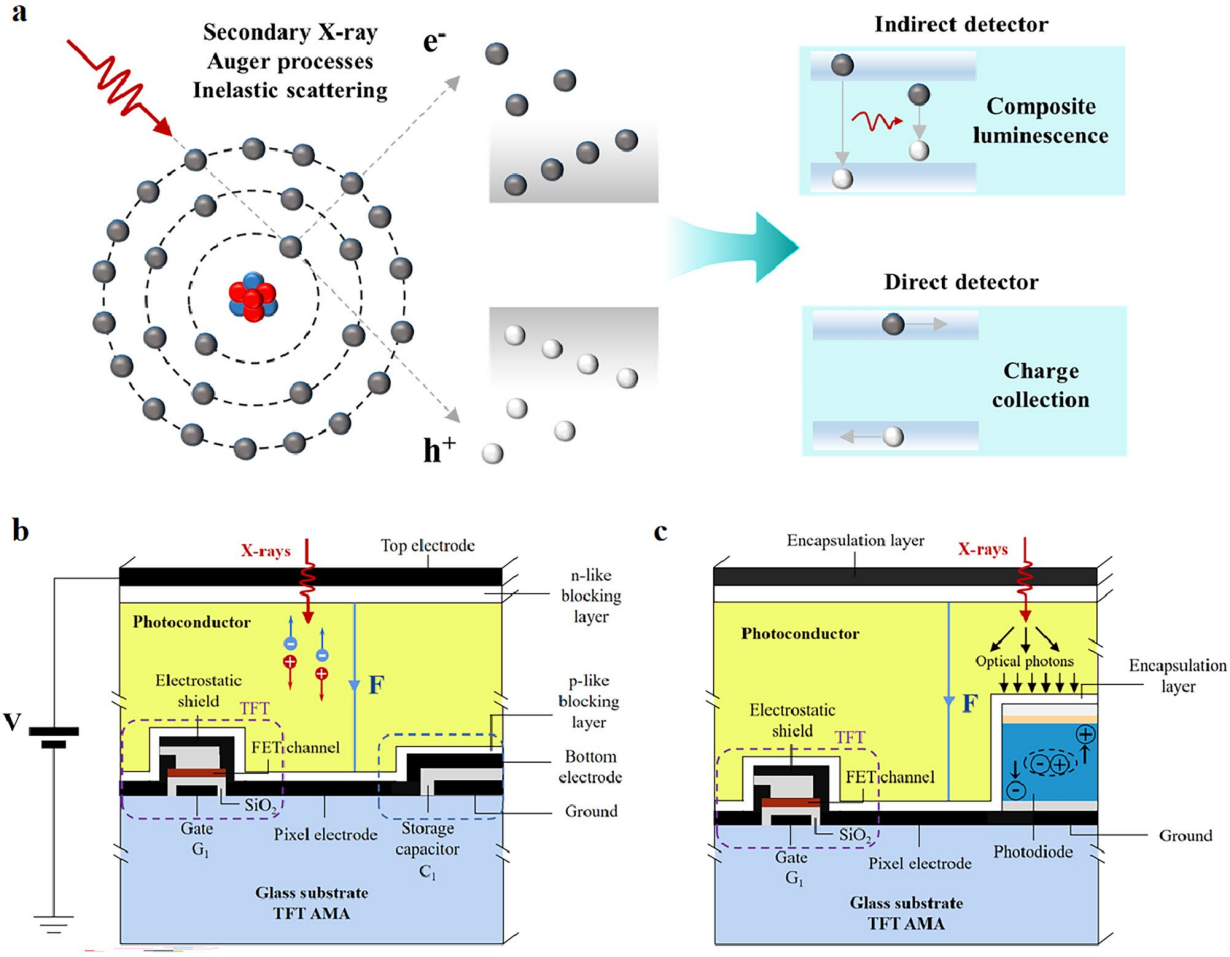
**a** Mechanism of indirect detectors and direct detectors for X-ray and particles; **b** Illustration of direct X-ray detection work; **c** Illustration of indirect X-ray detection work

The powerful penetrating capability of ionizing radiation has led to X-ray detection and imaging being used in a wide range of applications, including medical diagnosis, safety inspections, non-destructive testing, and scientific research [1–5]. At present, X-ray detection is mainly focused on direct current–current detection based on semiconductor materials and indirect detection based on the light–current of scintillators [6–13]. Scheme 1 shows the mechanism between X-rays and particles and the operating illustration of indirect detectors (scintillators) and direct detectors. Indirect detection typically uses scintillators to convert X-rays into visible light, which is then further collected and converted into an electric current by means of a photodiode or charge-coupled device. In contrast, direct detection converts X-rays directly into electrical charges and then stores and processes the collected charges into signals via a complementary metal oxide semiconductor (CMOS) array or a thin-film transistor (TFT). Direct detection avoids the scattering effect during visible light conversion and therefore offers the advantages of high detection sensitivity, excellent resolution, and rapid response speed. Hence in this mini-review, we primarily focus on direct detectors.

With the development of X-ray detection reaching its maturity, the performance requirements of detection materials have become more stringent. For the application of direct X-ray detection, although conventional semiconductors such as cadmium telluride (CdTe), amorphous selenium (α-Se), and silicon (Si) have been widely used, they suffer from problems such as insufficient mobility lifetime (μτ), poor absorption coefficient, and expensive manufacturing costs [14–16]. In this context, metal halide perovskite has arisen as an emerging alternative material in the field of superior performance X-ray detectors owing to its advantages such as large radiation absorption coefficients (containing heavy elements Pb, Bi, I, etc.) [17–20], high defect tolerance [21–23], large non-equilibrium carrier diffusion distances [24–26], and resistance to irradiation [27, 28].

For metal halide perovskites, the control of appropriate organic and inorganic components enables the adjustment of the dimensionality of the structure, resulting in the preparation of 3D, 2D, 1D and 0D perovskites. 3D perovskites are inherently environmentally sensitive and readily decompose in humidity, light and heat [29–33]. Furthermore, the lattice of 3D perovskite is relatively soft, which allows easy ion migration at low activation energy (*E*_g_) through the net of corner-sharing octahedra [34]. Meanwhile, compared to others perovskites, ion migration in 3D perovskites is not much of a hindrance. High ion migration will limit the charge extraction in the detector, thus affecting the device’s performance. At the same time, the ions at the interface will launch electrochemical reactions that lead to the irreversible degradation of the material, which will seriously damage the stability of the device [35, 36]. Relatively speaking, the relatively isolated structure of low-dimensional perovskites such as 0D can disrupt the channels for ion migration, while in-plane ion migration can be greatly restricted due to dielectric and quantum confinement. Hence 0D perovskites are usually considered to be stable [37]. However, the sensitivity of X-ray detectors derived from these 0D perovskites is noticeably lower than that of 3D perovskite because of their low charge mobility, which is particularly obvious in polycrystalline devices [38–40]. Fortunately, the presence of 2D perovskites provides an option to do both. The lead halide octahedral sheets in 2D perovskites are segregated by organic spacers, and the arrangements of alternating organic and inorganic layers produce a particular electronic structure called a 2D multiple quantum well (MQW) [41–43]. Compared with others perovskite, due to the existence of a large hydrophobic ammonium salt organic layer and MQW, 2D perovskite has natural advantages in the area of X-ray detection, such as high carrier mobility, suppressed ion migration for stable current output, excellent operation stability, flexible and adjustable structure [44]. Therefore, 2D perovskite has become one of the most bright materials for the next generation of X-ray detector technology.

In this mini-review, we introduce and enumerate the classification of 2D perovskite, summarize the manufacturing methods of 2D perovskite X-ray direct detectors, highlight their unique performance characteristics, and briefly discuss the application of 2D perovskite in scintillators. Finally, we analyze the problems it faces and present some of our insights.

## Classification of 2D Metal Halides

According to the binding mode of interlayer spacer cations, 2D perovskites can usually be classified into three types: The Ruddlesden-Popper (RP) type (structure of R_2_A_*n* − 1_B_*n*_X_3*n* + 1_), The Dion-Jacobson type (DJ) (structure of RA_*n* − 1_B_*n*_X_3*n* + 1_) and the alternating cation in interlayer space type (ACI) ((GA)A_*n*_B_*n*_X_3*n* + 1_, GA-guanidinium) [45]. The n indicates the number of inorganic octahedral layers, and R denotes the interlayer spacer cations. A is a monovalent small radius cation, B is a metal ion such as Pb^2+^, and Sn^2+^, and X denotes a halogen ion (X^−^ = Br^−^/I^−^). The spacer cation R in RP phases is monovalent, such as phenethylammonium (PEA), and butylamonium (BA) [31, 46, 47]. For DJ phases, the R ions are divalent, mainly 1,3-propanediamine (PDA) or 3-(aminomethyl)piperidinium (3AMP), etc. [48]. And for ACI phases 2D perovskites, guanidine (GA^+^) is the only reported cation that can form ACI (2D) structures [49].

**Fig. 1:**
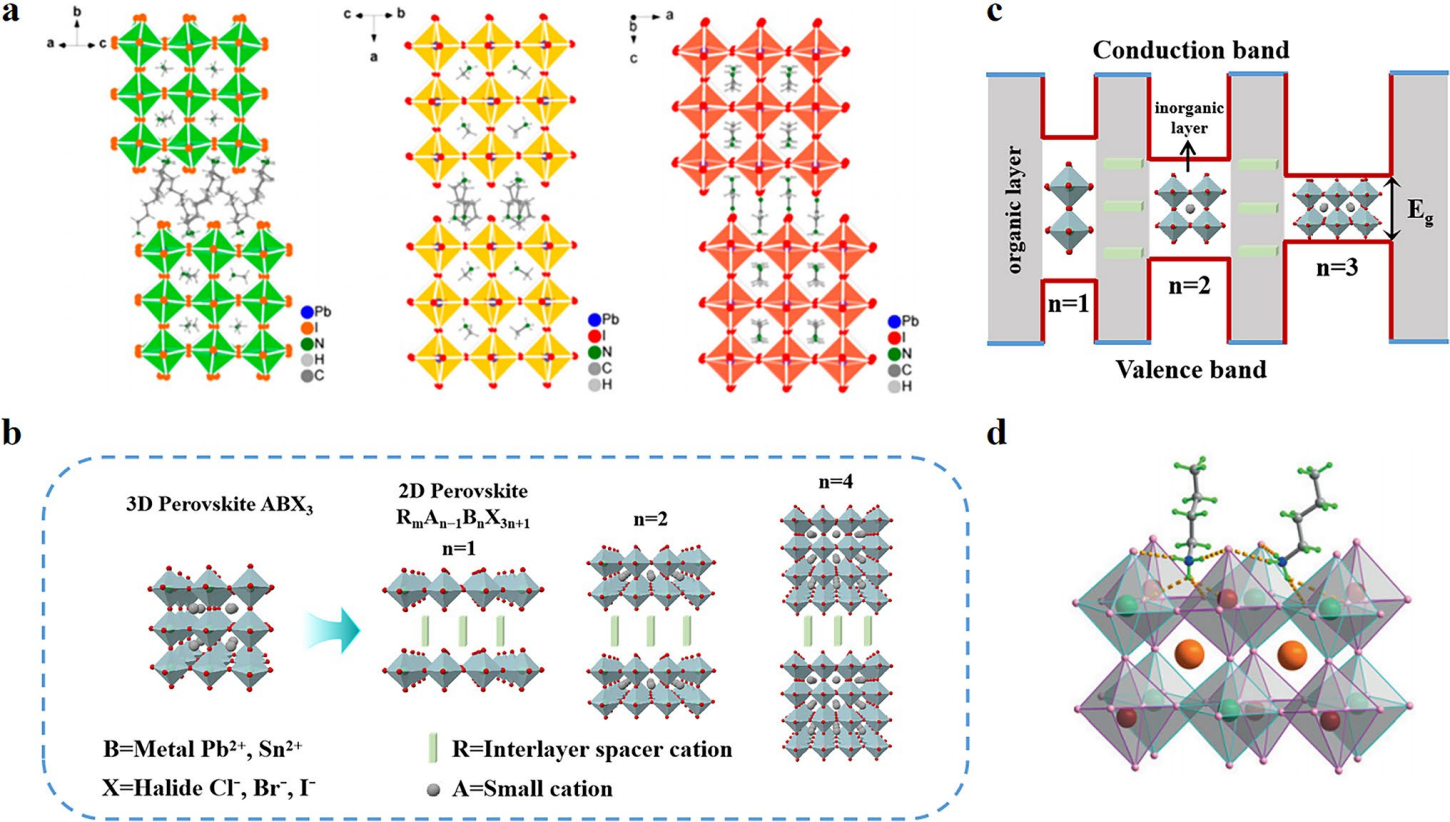
**a** *n* = 3 Schematic of crystal structure between RP phase, DJ phase, and ACI phase: (BA)_2_(MA)_2_Pb_3_I_10_; (3AMP) (MA)_2_Pb_3_I_10_; and (GA)(MA)_3_Pb_3_I_10_; Reproduced with permission [50]. Copyright 2019, American Chemical Society Publications; **b** Schematic diagram of the structure of 3D and 2D perovskites; **c** Quantum well structure of 2D perovskite; **d** Structural configuration of 2D double perovskite (BA)_2_CsAgBiBr_7_. Reproduced with permission [53]. Copyright 2019, Wiley–VCH Publications

Crystal structures of different 2D structure types and a comparison of 2D and 3D structures are shown in Fig. 1a, b [50]. In layered 2D perovskite, the inorganic octahedral layer serves as the potential well, and the number n dictates the width of the well and the band gap. In contrast, the large organic layer operates as the potential barrier, and its ionic radius can determine the width of the potential barrier (Fig. 1c) [42, 43]. Under this unique structure, RP-type, DJ-type, and ACI-type 2D perovskite have significant optical band gaps and small exciton binding energy to be used as active materials for optical detection [51].

In recent years, double perovskite structures have gradually entered the limelight owing to their low toxicity and excellent stability. The Cs_2_AgBiBr_6_ 3D double perovskite has well demonstrated the potential of lead-free perovskite in X-ray detection [52]. Thanks to the versatility and tunability of the double perovskite structure, the construction of 2D double perovskite becomes possible. In this context, an interesting 2D double perovskite structure comes into view. Z. Xu et al. [53] grew large-sized environmentally friendly 2D halide double perovskite (BA)_2_CsAgBiBr_7_ (BA = C_4_H_11_N) single crystals with the crystal structure shown in Fig. 1d. Two inhomogeneous octahedra of BiBr_6_ and AgBr_6_ are arranged alternately and orderly. The presence of the heavy element Bi ensures sufficient absorption of X-rays by the active layers, making it easier for carriers to be collected. Thus the X-ray detector derived from (BA)_2_CsAgBiBr_7_ single crystal achieves excellent sensitivity. It should be mentioned that compared to the three types of 2D perovskites, RP, DJ and ACI, 2D double perovskites are equivalent to modifying them to suit the environmental and other special requirements of perovskite X-ray detectors, rather than a completely new class of 2D perovskites.

## Preparation of 2D Metal Halide X-ray Detectors

In contrast to conventional semiconductors manufactured at elevated temperatures, 2D perovskites can be more easily manufactured at room temperature due to the relatively weak ionic bonding, effectively reducing the cost [54–58]. X-ray detection requires high photon attenuation and carrier mobility lifetime (μτ) product, so the preparation of materials is strict and harsh. To meet the requirements, many material preparation technologies have been developed lately. In this section, we review and summarize the processing technologies of 2D perovskites, mainly including the growth of perovskite single crystals and polycrystalline films’ preparation strategies, such as the hot-casting process, low-temperature blade-coating, and mechanical sintering process. In addition, we elaborate on some properties of perovskite materials in Table 1.

**Table 1:** Parameters of various 2D metal halides based direct X-ray detectors

Materials	Size	*μτ* product (cm^2^ V^−1^)	Sensitivity (μC Gy^−1^ cm^−2^)	Detection limit (μGy s^−1^)	References
(BDA)PbI_4_ SC	–	4.43 × 10^–4^	242	0.43	[61]
(BA)_2_PbI_4_ SC	− 15 × 5 mm^2^	4.5 × 10^–4^	148	0.241	[77]
BA_2_PbBr_4_ SC	160 μm thick	1.1 × 10^–5^	726.18	0.0082	[78]
(BA)_2_CsAgBiBr_7_ SC	10 × 10 × 3 mm^3^	1.21 × 10^–3^	4.2	–	[53]
(F-PEA)_2_PbI_4_ SC	10 × 10 × 2 mm^3^	5.1 × 10^−4^	3402	0.023	[79]
(BA)_2_CsPb_2_Br_7_ (ab plane)	–	–	13,260	0.0725	[31]
BA_2_EA_2_Pb_3_Br_10_ SC	2 mm thick	1.0 × 10^−2^	6800	5.5	[80]
(DFPIP)_4_AgBiI_8_ SC	6–10 mm^2^ × 2 mm	1.1 × 10^–5^	188	3.13	[81]
(DGA)PbI_4_ SC	–	4.12 × 10^–3^	4869	0.0954	[44]
(3AP)PbCl_4_ SC	5.2 × 2.6 × 1.4 mm^3^	2.74 × 10^–3^	791.8	1.54	[82]
PEA_2_PbBr_4_ film	1.9 ± 0.8 µm thick	1.09 × 10^–5^	806	0.042	[83]
(BA)_2_(MA)_2_Pb_3_I_10_ film	470 nm thick	–	2.76 × 10^5^	10	[84]
PEA_2_MA_8_Pb_9_I_28_ film	**–**	2.6 × 10^–5^	10,860	0.069	[36]

### Single Crystals

Perovskite single crystals have received publicity owing to their excellent properties, such as lower bulk defect density of states, longer carrier lifetime and diffusion length, and higher carrier mobility [59, 60]. 2D perovskite single crystals are generally grown by changing the process conditions to induce a change in solubility.

**Fig. 2:**
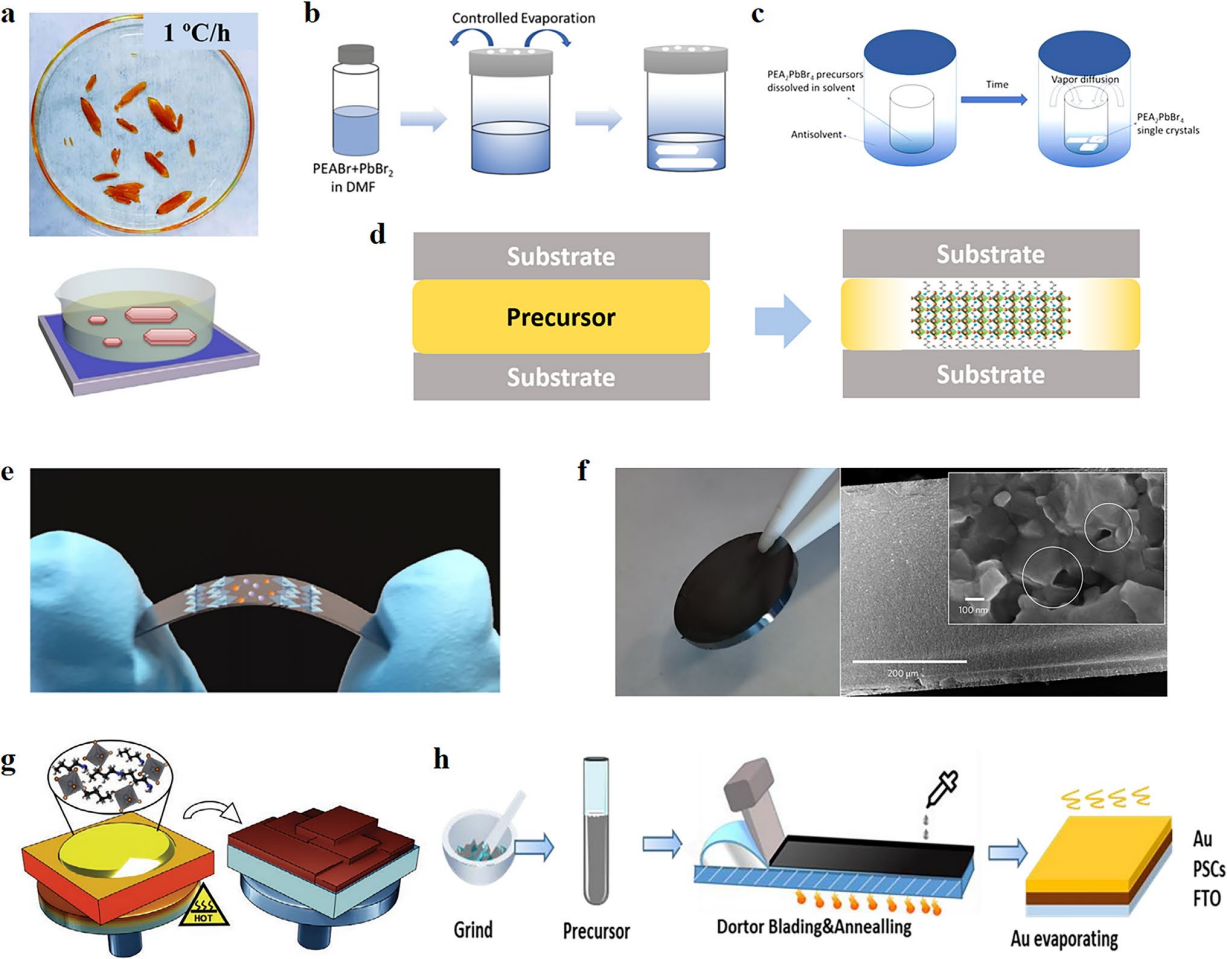
Methods for preparing 2D perovskite: **a** Photograph of (BDA)PbI_4_ crystals grown by the temperature crystallization method; Reproduced with permission [61]. Copyright 2020, Wiley–VCH Publications; **b** Schematic diagram of the process of growing (PEA)_2_PbBr_4_ single crystals by controlled evaporation; Reproduced with permission [62]. Copyright 2019, The Royal Society of Chemistry; **c** Schematic diagram of the process of growing 2D (PEA)_2_PbBr_4_ perovskite single crystals by modified anti-solvent vapor crystallization method; Reproduced with permission [63]. Copyright 2017, American Chemical Society Publications;** d** Schematic diagram of the synthesis of large-area 2D BA_2_MA_2_Pb_3_I_10_ (*n* = 3) perovskite single crystals by the space-constrained method; Reproduced with permission [64]. Copyright 2018, American Chemical Society Publications;** e** Optical image of the EDA(MA)_3_Pb_4_I_13_ flexible film under mechanical bending. Reproduced with permission [69]. Copyright 2020, American Chemical Society Publications;** f** Images and SEM cross-section of the sintered MAPbI_3_ wafer. Reproduced with permission [72]. Copyright 2017, Springer Nature Publications; **g** Schematic illustration of hot-casting process; Reproduced with permission [70]. Copyright 2022, Wiley–VCH Publications; **h** Schematic of the fabrication process of the perovskite thick film-based device through the low-temperature blade-coating method. Reproduced with permission [36]. Copyright 2021, Wiley–VCH Publications

The cooling crystallization method is a common strategy for the preparation of single crystals, which can change the solubility by cooling and thus induce nucleation crystallization of the solution. Simultaneously, the nucleus formation triggers a decrease in solution concentration and inhibits further nucleation. Therefore, uniform nucleation can be easily achieved by reasonably controlling the cooling rate, which can grow large single crystals. For example, Y. Shen et al. [61] successfully prepared centimeter-sized BDAPbI_4_ single crystals by cooling the precursor solution from 90 °C to room temperature at a rate of 1 °C h^−1^ by a modified cooling crystallization method (Fig. 2a). The evaporation crystallization is very similar to cooling crystallization, only differing in the scope of applications and operational steps. Evaporation crystallization mainly applies to materials whose solubility does not vary much with temperature. Zhang et al. [62] achieved the nucleation growth of crystals by evaporating the solvent to supersaturate the solute and preparing large-size (> 200 mm^2^) 2D (PEA)_2_PbBr_4_ perovskite single crystals (Fig. 2b). The anti-solvent method has attracted wide attention owing to its low cost and simple operation. It can manufacture high-quality single crystals through tuned nucleation and growth. The solvents used for antisolvent-assisted crystallization include one-component chlorobenzene (CB), toluene (TL), and ether, as well as mixed antisolvent systems, which help to regulate the crystal growth dynamics by optimizing the mixing ratio of the system. H. Tian et al. [63] synthesized 2D (PEA)_2_PbBr_4_ perovskite single crystals at room temperature using a modified anti-solvent vapor crystallization method (Fig. 2c). The anti-solvent CB vapor dispersion into the solution effectively reduces the solubility, resulting in easier nucleation and crystallization. The space-constrained method is a novel preparation method that can regulate the thickness of crystals to a certain extent and achieve large-area preparation. Xiao et al. [64] synthesized large-area 2D BA_2_MA_2_Pb_3_I_10_ perovskite single crystals using a space-constrained method. They used two non-wetting substrates to construct the confined space and inserted a saturated perovskite solution into the space (Fig. 2d). The single crystals were grown by the cooling-induced supersaturation method.

Notably, although the preparation of 2D perovskite crystals has made good progress nowadays, they are often prepared by controlling the molar ratios of different precursor substances. The molar ratios between different ions in the final formulation may be far from the stoichiometric ratios of the desired phases for pure phase substances with high n values. So it requires constant trial and error, which is rather tricky. Solving this problem by regulating the deposition process and optimising the preparation process may be a good option to stop multiphase formation [65, 66].

### Polycrystalline Films

Although 2D perovskite single crystals have demonstrated superior optoelectronic properties in the X-ray detection area, the preparation and processing of large-area single crystals are still challenging due to their brittleness. At the same time, single crystal cannot meet the needs of flexible devices because of its poor mechanical flexibility, which deprives it of the ability to adapt to non-planar and limits its application scenarios. Therefore, people have turned their attention to the preparation of thin films. Compared to single crystals, polycrystalline thin films offer the advantages of large-area fabrication, flexible applications and substrate integration [67, 68]. The spin-coating strategy to obtain large-area absorption layers is a conventional means of preparing thin films. Lai et al. [69] successfully prepared EDA(MA)_3_Pb_4_I_13_ flexible films by a simple one-step spin-coating process (Fig. 2e). The 2D perovskite device can be bent down to a radius of 2 mm, and after 10,000 cycles of bending tests, there was no significant performance degradation.

The absorption coefficient and thickness are key factors affecting the absorption of X-rays by the material, and thicker absorption layers are often required for ionizing radiation with strong penetrating capabilities. Therefore, for X-ray detection applications, thick, large absorption layers are often required to obtain sufficient X-ray absorption and to achieve imaging of large-area objects. The usual polycrystalline films are difficult to meet, hence the research on thick films is urgent and relevant for X-ray detection. The improvement based on spin coating is a popular method to prepare 2D perovskite thick film. At present, the common improvement measures are heating, ion engineering, additives, and so on. Tsai et al. [70] introduced n-butylamine iodide into methylammonium lead iodide precursor and cast it at elevated temperatures. Cation engineering by incorporating BAI in the perovskite precursor can enhance the degree of crystallinity. The elevated temperatures allow rapid solvent discharge during film formation, eliminating the solvent trapping problem. As a consequence, they achieved the preparation of dense polycrystalline 2D perovskite thick films of 10 μm on both rigid and flexible substrates (Fig. 2g). Due to the benefits of low-priced and great compatibility with substrate materials, the blade coating method is also a powerful tool for thick film preparation [71]. As shown in Fig. 2h, He et al. [36] prepared quasi-2D PEA_2_MA_8_Pb_9_I_28_ perovskite polycrystalline thick films using the low-temperature blade-coating method, which possesses the advantages of large grain size and inferior defect density. The X-ray detector based on it demonstrates a sensitivity of 10,860 µC Gy_air_^−1^ cm^−2^ with steady dark current and photocurrent response. Shrestha et al. [72] proposed a mechanical sintering process to prepare perovskite wafers with millimeter thickness (Fig. 2f). This method guides the preparation of efficient and low-priced 2D perovskite wafer X-ray detectors. Moreover, the development of strategies such as vacuum vapor deposition and composite films will also bring stronger competitiveness to thick film X-ray detectors [73–76].

The preparation of 2D perovskite thick film is an essential means to improve the photoelectric conversion efficiency of detectors, which is of great significance to its development. However, an increased thickness is often accompanied by an increased defect density and a reduced carrier diffusion length. Optimizing or achieving the balance between the two remains a challenge to be solved.

## Performance Characteristics of 2D Metal Halide X-ray Detectors

### Low Ion Migration

There are shallow electron and hole traps in the band gap of 3D perovskite, which can trap free carriers. When the bias voltage is applied, these trapped charges may release and induce field-driven ion migration. The accumulation of ion migration can directly cause changes in the built-in electric field of the perovskite and even the local crystal structure, which in turn can seriously affect the stability and optoelectronic performance of the device. So, higher ion migration has been a notorious problem in 3D perovskites [52, 85, 86]. For perovskite optoelectronic devices, a large number of ions will diffuse in 3D perovskite under the effect of the electric field. The migrated ions may corrode the metal electrodes on the surface, damage the device, and create many voids as the center of non-radiative recombination, resulting in a degradation of the photovoltaic performance. On the other side, “mobile ions” that accumulate at the device interface can migrate across the interface and react with it, affecting the device’s operating mechanism [86, 87]. Higher ion migration, especially for X-ray detectors, also leads to baseline drift problems. The relatively low resistivity and intense ion migration can dramatically increase the detector’s current drift and dark current noise, thus reducing the resolution and stability of the detector. Also, ion migration is a critical cause of photocurrent instability in detectors [40, 88].

**Fig. 3:**
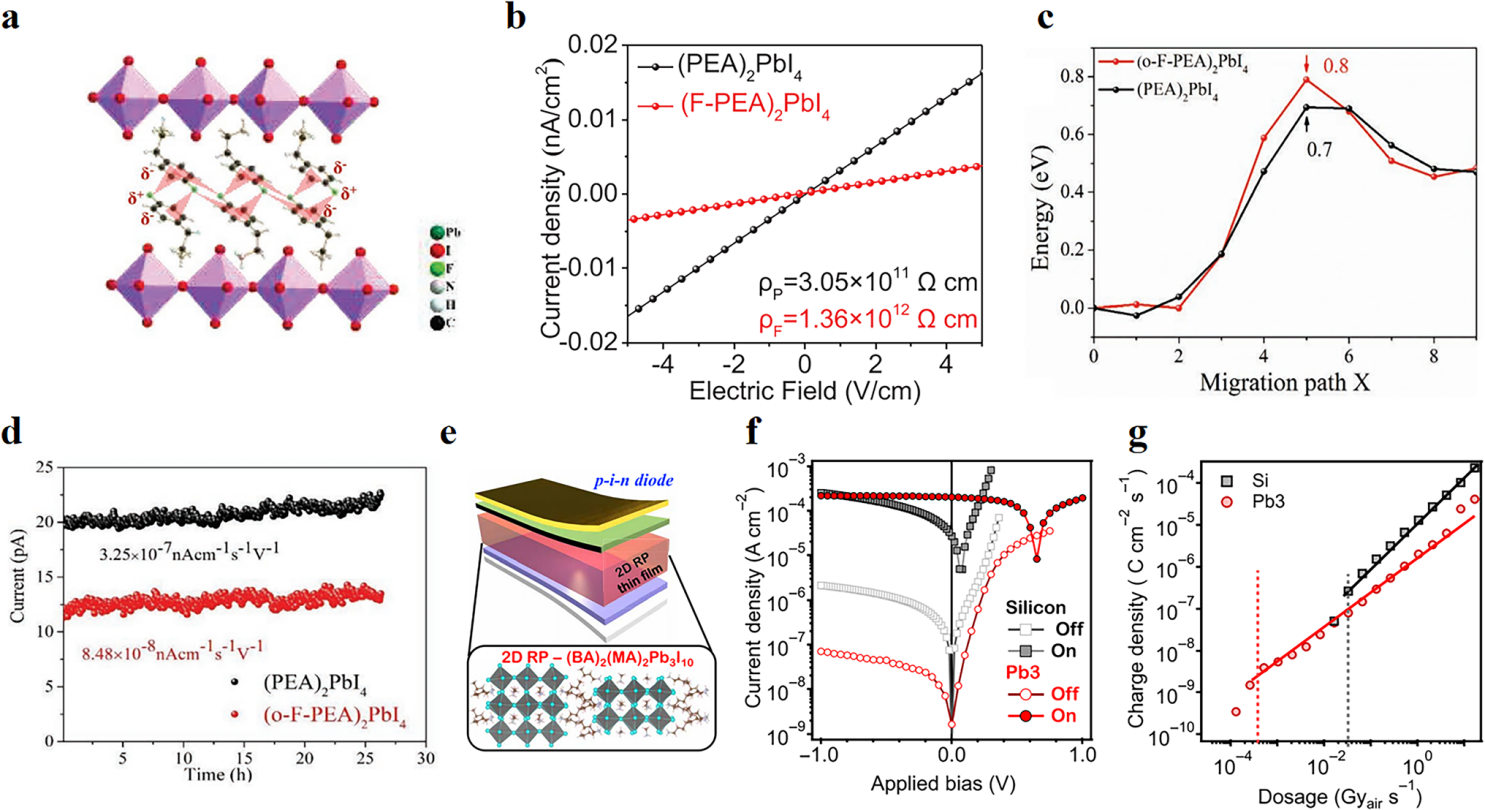
**a** Optimized single crystal structure of (F-PEA)_2_PbI_4_; **b** Resistivity of (PEA)_2_PbI_4_ and (F-PEA)_2_PbI_4_ single crystals; Reproduced with permission [79]. Copyright 2020, Wiley–VCH Publications; **c** AEIM of (o-F-PEA)_2_PbI_4_ with (PEA)_2_PbI_4_ calculated by DFT simulation; **d** Dark current measurements of (o-F-PEA)_2_PbI_4_ versus (PEA)_2_PbI_4_ (20 V, RH 60%); Reproduced with permission [91]. Copyright 2021, Wiley–VCH Publications; **e** Schematic illustration of the p-i-n thin film X-ray detector based on (BA)_2_(MA)_2_Pb_3_I_10_; **f** J–V characteristics of (BA)_2_(MA)_2_Pb_3_I_10_ (red) and silicon (black) reference devices in the dark and under X-ray (10.91 keV) exposure; **g** X-ray response currents of (BA)_2_(MA)_2_Pb_3_I_10_ and silicon diode at various dose rates under zero bias conditions. Reproduced with permission [84]. Copyright 2020, American Association for the Advancement of Science

The organometallic octahedra of 2D perovskites are separated by long organic spacer cations, which effectively prevent ion migration and can improve the sensitivity of the device to X-rays [64, 89]. Also, higher generation energy related to ionic vacancies in 2D perovskites in comparison to 3D perovskites facilitates ion migration suppression in layered perovskites [64, 90]. Besides, with the help of flexible tunability, the 2D perovskite structure still has much room to improve the inhibition effect of ion migration. Studies have shown that blocking ion migration paths and increasing the activation energy of ion migration are effective inhibition measures. H. Li et al. [79] introduced defective F atoms as supramolecular anchors in 2D perovskite organic spacer layers, which effectively suppressed the ion migration phenomenon by interrupting the ion migration path after anchoring. They prepared a 2D (F-PEA)_2_PbI_4_ perovskite single crystal hard X-ray detector with a bulk resistivity of 1.36 × 10^12^ Ω cm (Fig. 3a, b), which resulted in low device noise for hard X-ray detection. Zhang et al.[91] overcame ion migration by enhancing chemical bonding, shortening the distance between adjacent organic cations in the lattice by incorporating fluorine atoms into the neighboring positions of phenethylamine to enhance electrostatic interactions between F atoms and adjacent benzene rings. The activation energy for ion migration (AEIM) of (o-F-PEA)_2_PbI_4_ single crystals was increased in comparison to that of (PEA)_2_PbI_4_ single crystals (Fig. 3c). The improved AEIM also enhances thermal stability. As a consequence, the dark current of the (o-F-PEA)_2_PbI_4_ 2D perovskite single crystal X-ray detector is decreased by a factor of 2 compared to (PEA)_2_PbI_4_ and by a factor of 4 at 20 V bias (Fig. 3d). Besides, reducing the defect density and adjusting the number of perovskite layers n would be effective strategies to improve ion migration [27, 90].

From the perspective of external conditions, researchers usually tend to apply a high electric field to optimize detection properties. Still, a high electric field is often accompanied by high dark current and ion migration. Recently, self-powered photodetectors have received great attention because they can operate without applied bias, effectively reducing the field-driven ion migration effect, suppressing device noise, and greatly improving portability [92]. 2D layered perovskite has attracted researchers’ interest in self-powered devices due to its structural flexibility. Tsai et al. [84] designed a novel 2D RP phase layered perovskite (BA)_2_(MA)_2_Pb_3_I_10_ X-ray detector (Fig. 3e). When exposed to the X-ray source, this 2D RP device demonstrates a significant increase in X-ray-induced current density at zero bias (Fig. 3f). The detector obtained a sensitivity of 0.276 C Gy_air_^−1^ cm^−2^ at zero bias. The sensitivity can be obtained by multiplying the slope of the linear region in the charge density–dosage–dependent plot (Fig. 3g) by the active layer thickness.

### Improved Charge Transfer Performance

The introduction of organic cations, although providing better stability and lower ion migration, greatly inhibits carrier transport due to the blocking of the organic layer, leading to larger charge transport anisotropy, increasing the charge accumulation on the surface and limiting its device performance [31, 93]. What is exciting is that the unique structure of 2D perovskite provides flexible improvement measures for it. In recent years, research on its anisotropic charge transport has made good progress. The current research focuses on: (1) inorganic layer thickness n; (2) organic spacer engineering; (3) lattice modulation; (4) crystal orientation, etc.

**Fig. 4:**
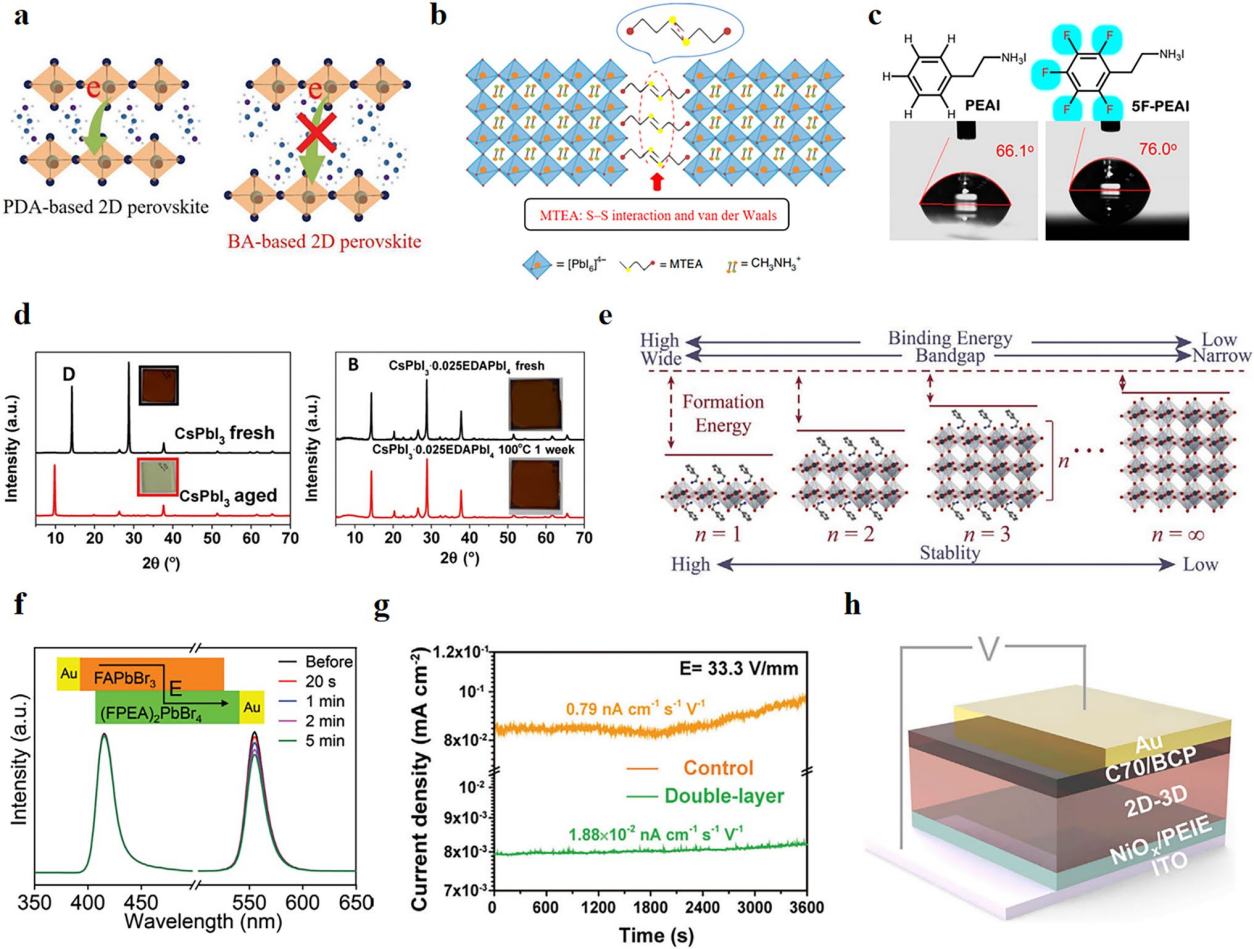
**a** PDA- and BA-based charge transport between inorganic layers in 2D perovskites; Reproduced with permission [95]. Copyright 2018, Wiley–VCH Publications; **b** Schematic crystal structure of (MTEA)_2_(MA)_*n* − 1_Pb_*n*_I_3*n* + 1_. Reproduced with permission [103]. Copyright 2020, Springer Nature Publications; **c** Chemical structure of PEAI and 5FPEAI. Contact angle of perovskite films treated with and without 5FPEAI. Reproduced with permission [104]. Copyright 2022, American Chemical Society Publications; **d** Phase stability of CsPbI_3_ in the presence and absence of EDAPbI_4_; Reproduced with permission [105]. Copyright 2017, American Association for the Advancement of Science; **e** Relationships between binding energy, band gap, formation energy, stability and dimensionality of low-dimensional perovskite. Reproduced with permission [107]. Copyright 2020, Wiley–VCH Publications. **f** PL spectra of 3D/2D film at different intervals under 1 V µm^−1^. Reproduced with permission [110]. Copyright 2021, Wiley–VCH Publications; **g** Temporal baseline tracking of the detectors made of the control (MAPbI_3_) and the double-layer perovskite film. Reproduced with permission [111]. Copyright 2021, Wiley–VCH Publications;** h** Schematic diagram of the device structure of 2D-3D perovskite-based X-ray detector. Reproduced with permission [113]. Copyright 2022, Elsevier Inc Publications

K. Wang et al. [94] reported a strategy for the rapid synthesis of 2D perovskite (BA)_2_(MA)_*n* − 1_Pb_*n*_I_3*n* + 1_ single crystal membranes and investigated the effect of inorganic layer thickness on transport anisotropy. The results show that increased inorganic layer thickness increased mobility and decreased anisotropy. Reasonable adjustment of the spacer layer can affect the electronic coupling between adjacent organic cations and barrier height, thus regulating the degree of crystal anisotropy. C. Ma et al. [95] reported a method to adjust the length of the organic spacer cation to adjust the anisotropy. They achieved significant charge transport enhancement by replacing the larger organic spacer cation butylammonium (BA) with the smaller propane-1,3-diammonium (PDA), which reduced the distance separating the inorganic perovskite layers (Fig. 4a). Xu et al. [96] constructed a binary spacer layer by adding 20% GA to F-PEA_2_MA_3_Pb_4_I_13_ and prepared smoother films with better vertical orientation and larger grains. Adding GA effectively accelerates the charge transfer and inhibits the non-radiative recombination in the films. Besides, carrier mobility is affected by the interaction between charge carriers and lattice vibrations (phonons). Therefore, it is also an excellent measure to improve carrier transport and stability by weakening the electron–phonon coupling through lattice distortion and the increase of hydrogen bonding, thus suppressing the disordered scattering of carriers [44, 97]. Changing the orientation of the quantum well (QW) to be perpendicular to the electrode and confining the free carriers in the QW can avoid the obstruction of charge transport by the organic layer. Hence, improving the charge transport in 2D perovskites by adjusting the crystal orientation has been the research focus in recent years. In response, Chen et al. [98] investigated the nucleation and growth process of 2D QWs, and also summarized the growth mechanism of 2D QWs, which is essential for the application of 2D perovskites in X-rays.

In addition to the transport issues, due to dimension and dielectric limitation, carriers confined in 2D perovskite QWs will produce high exciton binding energy and expand the formation of excitons instead of free carriers, which restrict their application in devices that require charge separation. Adding suitable additives may be an effective way to solve this problem, Gelvez-Rueda et al. [99] achieved the efficient formation of free mobile carriers by adding strong electron acceptor groups (perylene diimide organic chromophores) onto the surface of 2D perovskite nanosheets. The yield of these free carriers is ten times higher than in the absence of the acceptor, and the lifetime is tens of microseconds higher (two orders of magnitude). This scheme provides an effective concept for solving the charge separation problem. Moreover, changing the value of n reasonably to improve charge separation is also advisable. It has been shown that when *n* > 2, the exciton is separated into free carriers by the low energy state at the edge of the layer of the QW. This is beneficial to the photoelectric properties of 2D perovskite [100].

### Stability

Stability has been a stumbling block to the commercialization of perovskite X-ray detectors. 2D structures formed through dimensionality reduction are currently a successful solution to device stability. The large hydrophobic organic cations in 2D perovskites are spatial barriers for surface water adsorption and protect the fragile halide perovskite lattice [51, 101]. Hence the significant enhancement of water stability is a distinct advantage of 2D perovskites. Simultaneously, the moisture stability of 2D perovskites can be readily adjusted by changing the organic cations. For example, replacing hygroscopic organic cations with hydrophobic organic cations can enhance the stability of 2D perovskites in atmospheric environments. Zheng et al. [102] investigated the stability of four 2D perovskites under wetting conditions. The results show that (C_6_H_5_CH_2_NH_3_)_2_(FA)_8_Pb_9_I_28_ has the highest humidity stability compared to other 2D perovskites, and the device still has good optoelectronic performance when placed at 80% relative humidity for 500 h due to the strongest hydrophobicity from C_6_H_5_CH_2_NH_3_^+^. In addition, the selection of organic spacer cations that enhance the interaction of interlayer molecules can also improve moisture stability. H. Ren et al. [103] synthesized (MTEA)_2_(MA)_4_Pb_5_I_16_ (*n* = 5) perovskites (Fig. 4b), in which, besides the weaker van der Waals interaction, there is also an interaction between sulfur atoms in the two MTEA molecules, which effectively enhances the water stability of the perovskites. Recently, surface treatment is also used to improve moisture stability, H. Tsai et al. [104] found that the instability under voltage bias is immediately relevant to the humidity content in the environment. Coating the hydrophobic molecule 5F-PEAI on the 2D perovskite surface can inhibit the migration and degradation of ions, enabling the device to maintain good operating stability and low dark current even in a humid environment and under high voltage bias (Fig. 4c).

Furthermore, 2D perovskites also have excellent structural stability. By introducing bulky organic cations, the spatial site resistance between adjacent inorganic layers can be enhanced, thus improving the structural stability of 2D perovskites and effectively inhibiting the formation of non-perovskite phases. T. Zhang et al. [105] found that a few 2D (EDAPbI_4_) perovskites containing ethylenediamine (EDA) cations can stabilize α-CsPbI_3_, thus avoiding the formation of non-perovskite δ phases. The stability comparison with and without EDAPbI_4_ is shown in Fig. 4d. In addition to organic cations, the n value also affects the stability of 2D perovskites. 2D perovskites with lower n usually exhibit higher structural stability thanks to their higher generation energy (Fig. 4e) [106, 107].

It is worth mentioning that DJ perovskite is more stable than RP perovskite in terms of stability alone. There is a van der Waals gap between adjacent inorganic layers in RP perovskite because of the presence of two monovalent spacer cations. This gap not only hinders the charge transfer but also leads to water penetration. The diammonium in DJ perovskite, as a large spacer ion, not only can shorten the distance between inorganic frameworks but also form terminal hydrogen bonds with inorganic layers at both ends to avoid gaps between two adjacent inorganic layers, resulting in tighter connections, and more favorable charge transfer between inorganic flakes [108, 109].

### 2D/3D Heterojunctions

Perovskite materials of different sizes have demonstrated huge potential for direct X-ray detection, but each has inherent restrictions. The sensitivity of 2D perovskites is limited by poor carrier transport, while ion migration in 3D perovskites leads to baseline drift problems, and their stability has not been effectively addressed. To effectively combine the strengths of both 2D and 3D structures and enhance the performance of the material, 2D/3D bilayer perovskite stacking has been investigated. In this new design paradigm, the 3D layer ensures fast carrier transport, while the 2D layer mitigates ion migration, thereby improving both device efficiency and stability. He et al. [110] constructed single crystal heterojunctions of FAPbBr_3_/(FPEA)_2_PbBr_4_ through a simple solution-treated epitaxial growth method. Compared with 3D/3D structure, 2D perovskite PL hardly degraded in 3D/2D structure. The intensity of 3D perovskite PL remained above 80% under the same polarization conditions, indicating that the ion migration effect was inhibited in 3D/2D structure (Fig. 4f). In addition, the 3D/2D structure shows a lower defect density than the 3D structure. Xu et al. [111] developed an innovative X-ray detector based on 2D/3D bilayer perovskite films using an aerosol-liquid–solid process, in which 2D (PEA)_2_MA_3_Pb_4_I_13_ layers parallel to the substrate are cascaded with 3D MAPbI_3_ layers grown vertically. The presence of the 2D layer mitigates ion migration, providing a very stable baseline (Fig. 4g). Additionally, the 2D layer can improve the resistivity of the thin film without affecting the carrier extraction, and simultaneously expand the energy barrier of hole injection. These excellent properties result in an exceptional sensitivity of its device (1.95 × 10^4^ μC Gy_air_^−1^ cm^−2^). The construction of a heterojunction generally results in a built-in electric field, which often has a wide range of applications in self-powered devices. Zhang et al. [112] report a lead-free halide perovskite heterocrystal, (BA)_2_CsAgBiBr_7_/Cs_2_AgBiBr_6_. With its built-in potential, the device is capable of spontaneous charge separation/transport, while offering excellent sensitivity and stability.

In addition to the construction of stacked layers, a method of ion-exchange-induced slow crystallization (IESC) was recently reported by Peng et al. [113] They prepared devices on 2D-3D perovskite thick junctions by optimizing the process conditions, such as 2D and 3D precursor co-mixing ratios, and its structure is shown schematically in Fig. 4h. The optimized (BA_2_PbBr_4_)_0.5_-FAPbI_3_ X-ray detector demonstrated an excellent sensitivity of 1.36 × 10^4^ μC Gy_air_^−1^ cm^−2^, which shows huge capacity in X-ray imaging. The concern is that the introduction of 2D/3D structure not only improves the performance but also has obvious shortcomings. People need to consider and resolve how to achieve dense contact at the interface and improve the electronic coupling at the interface.

## 2D Metal Halide Scintillators

**Fig. 5:**
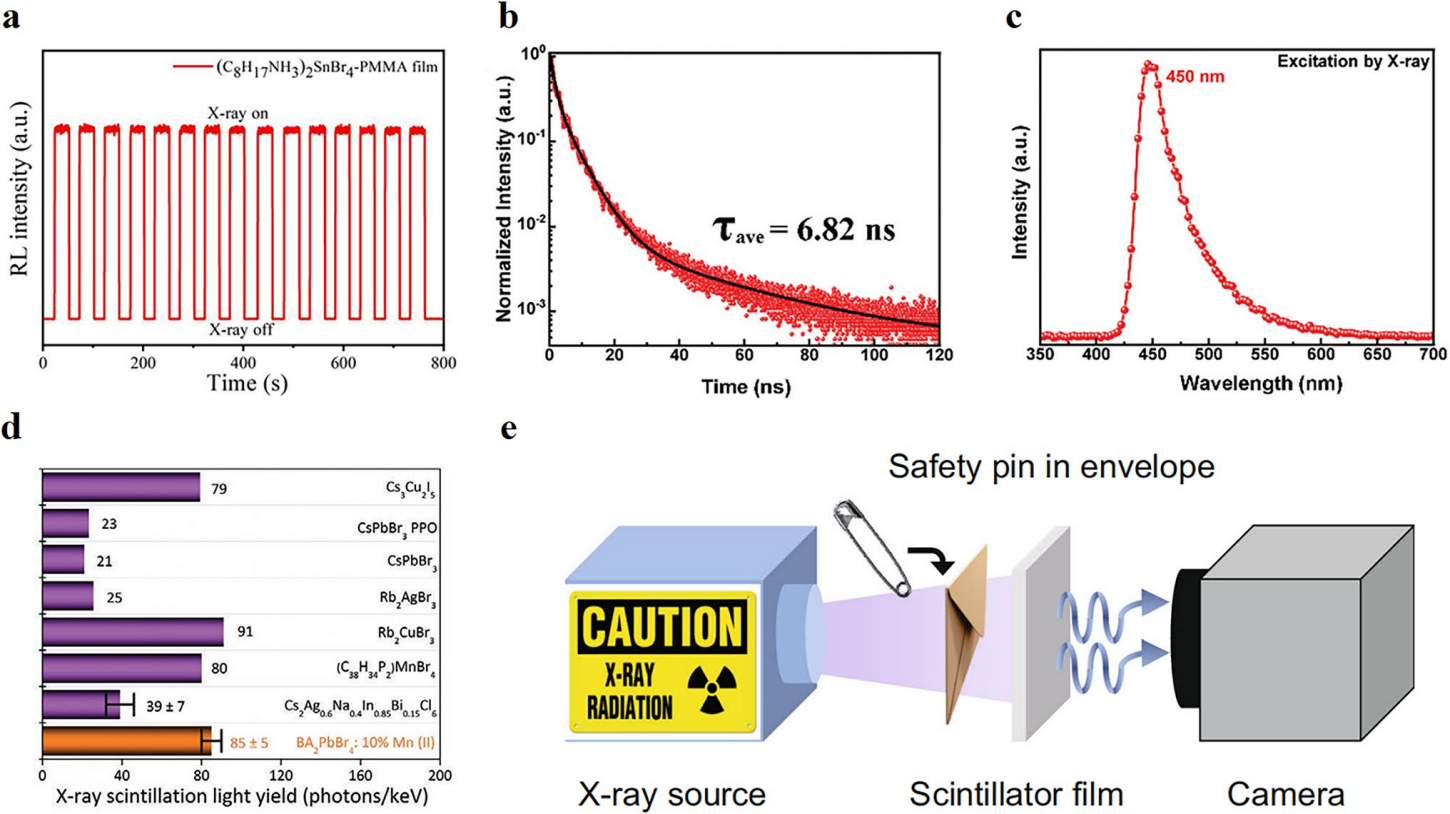
**a** Stability characterization of RL response of composite films at 40 kV. Reproduced with permission [114]. Copyright 2020, American Chemical Society Publications; **b** Time-resolved photoluminescence (TRPL) spectrum of BA_2_PbBr_4_; **c** Radioluminescence spectrum (RL) of BA_2_PbBr_4_. Reproduced with permission [65]. Copyright 2022, The Royal Society of Chemistry and the Chinese Chemical Society; **d** Comparison of the X-ray scintillation light yield. Reproduced with permission [115]. Copyright 2022, Wiley–VCH Publications; **e** Schematic of the experimental setup for the X-ray imaging system using 1:1 Li-(PEA)_2_PbBr_4_ as a scintillator. Reproduced with permission [116]. Copyright 2020, Springer Nature Publications

Due to their flexibility and lower price, indirect X-ray detectors are still mainstream in the detection market. Due to the MQW structure, 2D perovskite has excellent photoluminescence quantum yield and good stability, making it a potential scintillator material. Manufacturing cost, toxicity, X-ray conversion efficiency, and resolution are important criteria for judging scintillator materials. Based on the above criteria, Cao et al. [114] synthesized lead-free 2D (C_8_H_17_NH_3_)_2_SnBr_4_ perovskite scintillator in air environment. Its quantum yield is as high as 98% and has excellent stability and RL intensity under X-ray (Fig. 5a). Generally speaking, the number of inorganic layers n is difficult to control, so the 2D perovskite prepared in general is a quasi-2D, 2D, and 3D mixed phase. The preparation of pure 2D perovskite has always been a technical difficulty to be solved urgently. Recently, Xu et al. [65] prepared pure BA_2_PbBr_4_ single crystal by simple cooling crystallization method, which showed intense radioluminescence and ultrafast fluorescence lifetime (Fig. 5b, c). Introducing metal ions (e.g., Mn^2+^, Li^+^, Sr^2+^, Ba^2+^) into the inorganic layer is a common method to improve the light yield of the scintillator. W. Shao et al. [115] introduced an efficient manganese (II) activated 2D BA_2_PbBr_4_: Mn (II) perovskite. With an appropriate amount of Mn (II) dopant as an activator, the luminescence properties of BA_2_PbBr_4_ were optimized by effective energy transfer. The device based on the optimum BA_2_PbBr_4_: 10% Mn (II) perovskite exhibits excellent sensitivity and light yield as high as 85 ± 5 photons keV^−1^ (Fig. 5d). Moreover, Li-doped 2D (PEA)_2_PbBr_4_ perovskite crystals were prepared by Xie et al. [116], in which Li-dopant serving as traps can broaden the X-ray luminescence and enhance the intensity. The light yield of 1:1 Li-doped (PEA)_2_PbBr_4_ crystal is 11,000 ± 500 photons MeV^−1^, which is much higher than that of undoped crystal 8000 ± 800 photons MeV^−1^. Finally, they also demonstrated X-ray phase-contrast imaging of Li-(PEA)_2_PbBr_4_ as a scintillator film (Fig. 5e).

## Summary and Outlook

In summary, 2D perovskites are a novel and promising category of materials that can complement 3D perovskites for X-ray detection and other optoelectronic applications owing to their elevated stability and tunability of performance through the appropriate design of organic spacer cations. In this mini-review, the synthesis scheme of 2D perovskite is introduced. Then the research status of 2D perovskite in the field of direct X-ray detectors is illustrated through its performance analysis. Finally, we discuss its application to scintillators. To further advance 2D perovskite X-ray detectors into the market, we also point out key challenges to be explored. We hope that these insights will guide us in optimizing the capabilities of 2D perovskite X-ray detectors:

### Material Coupling

Owing to the insulating effect of organic spacer cations, 2D perovskites usually have high charge transport in the inorganic plane and inferior cross-layer transport, which severely degrades their electrical properties. Moreover, the charges in the compounds tend to recombine, thus significantly reducing the photoelectric conversion performance of the devices. Although the introduction of 3D layers can improve the transmission problem, the 2D/3D interface problem due to dielectric mismatch is still a pressing research challenge to be solved. Moreover, designing organic cations with strong charge transfer capability or combining them with other 2D materials (Graphene and its derivatives, black phosphorous, transitional metal dichalcogenides, and transition-metal carbides and nitrides) may be an effective solution [117–119].

### Lattice Adaptation

The unique layered design of 2D perovskite gives flexibility to its structure. The selection of organic spacer cations and halides, and the variation of perovskite layer thickness help to adjust the properties of 2D perovskite. However, the introduction of different spacer layers in 2D perovskites will lead to different degrees of lattice deformation, which is a difficult process to control. Therefore, research is also needed to discover more new materials with good lattice fitness and excellent performance of spacer layers.

### Toxicity and Synthesis Optimization

2D perovskites generally contain the toxic metal Pb, which has been criticized. For now, using Sn or constructing a double perovskite structure using two metals instead of Pb is a better choice to solve the toxicity. Meanwhile, the manufacturing process of 2D perovskite needs to be designed rationally to improve device performance and reduce costs.

In conclusion, although the environmental stability and ion migration properties of 2D halide perovskites are better than those of 3D devices, their optoelectronic performance is still unsatisfactory, and there is still considerable scope for improvement. In the area of X-ray detection, there is a long way to go to replace the current commercial materials for wider commercial applications, both in 2D and even in all halide perovskites.
